# Prevalence of Malnutrition and Its Associated Factors Among Children in the 5–15 Years Age Group: A Community-Based Cross-Sectional Study From Selected Rural Areas of Khordha District, Odisha

**DOI:** 10.7759/cureus.105874

**Published:** 2026-03-26

**Authors:** Rekha Singh, Geetarani Nayak, Amit Satapathy

**Affiliations:** 1 Pediatric Nursing, All India Institute of Medical Sciences, Bhubaneswar, Bhubaneswar, IND; 2 Pediatrics, All India Institute of Medical Sciences, Bhubaneswar, Bhubaneswar, IND

**Keywords:** associated factor, children and adolescent, malnutrition, prevalence, stunting and undernutrition

## Abstract

Introduction

Malnutrition is one of the most common causes of morbidity and mortality among children and adolescents throughout the world. Various factors, including socio-demographic characteristics, poor feeding practices, and childhood diseases, contribute to malnutrition. This study aimed to assess the prevalence of malnutrition and its associated factors among 5 to 15-year-old children of selected rural areas of Khordha District, Odisha.

Method

A community-based descriptive cross-sectional study was conducted among children in Simlipatana and Kujjimahal villages of Khordha district, Odisha. All eligible children (5 to 15 years of age) residing in both study areas were included in the sampling frame. A total of 380 participants were enrolled in the study, based on a 45% prevalence, using simple random sampling. Data were collected using participants' socio-demographic characteristics, anthropometric measurements, the IAP growth chart app, and a validated questionnaire to assess factors associated with malnutrition. The data were analysed using SPSS software, version 26.0 (IBM Corp., Armonk, NY).

Results

The study findings revealed that the prevalence of malnutrition was 41.3%, with 13.9% underweight, 12.9% overweight, 5.3% obese, and varying degrees of stunting. Among malnourished children, 56.32% were female, and nearly half (43.67%) were male. Malnutrition was significantly associated with sociodemographic factors like family type, income, parental education, and hygiene practices. Dietary intake patterns, such as consumption of milk, pulses, and fruits. Logistic regression analysis revealed that four key significant factors were associated with malnutrition of children, including large family size (odds ratio (OR) = 2.019, P < 0.003), poor hygiene (OR = 2.082, P < 0.006), lack of parental education (OR = 2.045, P < 0.003), and inadequate dietary intake (OR) = 2.115, P < 0.011).

Conclusions

The overall prevalence of malnutrition was 41.3%, and it decreased with age. Dietary patterns and sociodemographic profiles are significantly associated with the development of malnutrition.

## Introduction

Malnutrition remains a significant public health issue globally, leading to stunted growth, diminished cognitive development, and heightened vulnerability to infectious diseases in children [1]. Globally, an estimated 149 million under-fives are stunted, and 45 million are wasted, reflecting the magnitude of the problem in low- and middle-income countries (WHO), and are the cause of at least half of all childhood deaths, and one-third of child deaths are due to undernutrition only [2]. It accounts for 11% of the global disease burden. It is more prevalent in countries with low and lower-middle economic status [3]. India’s children are underweight, which is the highest in the world, and is almost double that of Sub-Saharan African countries [4].

Undernutrition comprises four forms: stunting (low height-for-age), wasting (low weight-for-height), underweight (low weight-for-age), and micronutrient deficiencies. India, a developing country, covers 40% of the world's undernourished children [5]. A cross-sectional study conducted among 150 school children aged 6-19 years in government schools in Kashmir reported that 34.7% were underweight, 3.3% were overweight, and 3.3% were obese, with an overall malnutrition prevalence of 41.3% [6].

The National Family Health Survey-5 (2019-2020) reports that 35%, 19.3%, and 32.1% of children younger than five years were stunted, wasted, and underweight, respectively. This suggests that nutritional deficits frequently persist beyond early childhood into later school ages, indicating the need for sustained intervention. Mostly rural populations are disproportionately affected due to poverty, limited dietary diversity, and gender bias. Despite government initiatives, such as the Integrated Child Development Services (ICDS), mid-day meal schemes, and the Prime Minister's Overarching Scheme for Holistic Nutrition (POSHAN), the persistence of undernutrition in India is impeding efforts to achieve the targets set by the National Nutrition Mission, which aims to reduce the stunting in children (0-6 years of age) from 38.4% to 25% by 2022 (Mission 25 by 2022) in India [7].

Most of these programmes focus on younger children and pregnant/lactating mothers, rather than leaving gaps in coverage for older children. School-aged children (5-15 years) are often overlooked in nutritional surveillance compared to children under five years of age. Evidence reports that nutritional deficits frequently persist beyond early childhood into later school age. This has inadvertently led to overlooking the nutritional status of school-age children. Older children and adolescents are at greatest risk of undernutrition because they experience a growth spurt, which increases their need for essential micronutrients to support the body's increased demands during puberty and development. Studies have reported that undernutrition is associated with delayed sexual maturation, which is required to reach adult developmental potential [8,9].

Malnutrition adversely affects physical growth, cognitive development, immunity, and academic performance. Globally, millions of children are affected by nutritional deficiencies, which contribute significantly to childhood morbidity and mortality. It can lead to fatigue, poor concentration, and illness, resulting in higher absenteeism and school dropout rates [8]. Consistently, Odisha has been ranked with elevated levels of child malnutrition among the states, especially in rural and tribal areas, where poverty, food insecurity, and limited healthcare access prevail.

Despite government initiatives, such as the Midday Meal Scheme, malnutrition persists among school-aged children. Khorda District in Odisha presents a unique context in which rural communities face challenges such as low household income, inadequate sanitation, and traditional dietary practices. Many studies are conducted in urban or hospital settings, while rural communities remain underrepresented. There is limited evidence documenting the prevalence and determinants of malnutrition among school-going children in selected rural areas of Odisha.

Considering the limited data on school-age children (5-15 years) in rural areas, particularly in Odisha, there was a need to assess the prevalence of malnutrition and its associated factors among school-going children in a selected rural area of Khorda District, Odisha. The findings will help develop targeted nutritional interventions, improve school health programmes, and guide policymakers to address malnutrition in this vulnerable age group, thereby improving child health and nutrition in similar rural settings in India.

## Materials and methods

A community-based cross-sectional study was conducted in Simlipatana (population: 2,293; 471 households) and in Kunji Mahal village (population: 1,684; 374 households), both located in Chandaka Tehsil of Khorda District, a rural and urban field practice area under the aegis of All India Institute of Medical Sciences (AIIMS), Bhubaneswar, during November-December 2020.

The list of 5-15-year-old children residing in both villages was collected from Accredited Social Health Activists (ASHA) workers, and the total number of eligible participants based on the inclusion criteria was recorded. Study participants were selected using simple random sampling with a random number table and enrolled in the study. The multipurpose health worker verified the list. On the day of the visit, if the study subject was not present, they were covered in the next visit. If, on three consecutive visits, a particular house was found to be locked, the study subject was dropped from the study, and the next eligible subject was considered. The required sample size was calculated using the cross-sectional study formula, based on a previous prevalence of malnutrition among adolescents (p = 45%) [10]. At a 95% confidence level and 5% margin of error, the following formula was used: N = Z²(1 - α/2) x P(1 - P)/d², where, N = sample size, ​​​​​​​Z(1-α/2) = 1.96, the value of the standard normal variate corresponding to the level of significance α of 0, d = specified absolute error on either side of the mean; P = prevalence of malnutrition. Substituting Z = 1.96, p = 45, q = 55, d = 5%, the final sample size was 380.

A total of 420 houses were surveyed, and 380 were enrolled in the study. The response rate was (380/420 =90.5%). The reasons for non-participation were (40 households excluded/locked). The inclusion criteria were as follows: 5-15-year-old children residing in Simlipatana and Kuji mahal villages, available for data collection, and able to understand Hindi/English/Odia. The exclusion criteria were as follows: Children suffering from congenital heart disease, known cases of TB, HIV, or cancer. 

The data collection tool consists of four sections. Section A contains a structured questionnaire with 17 closed-ended items to assess participants' and their families' socio-demographic details. Section B contains an anthropometry measurement form with three items: weight was measured in kg, height in cm, and BMI in kg/m². A calibrated digital weighing machine from the White Cherry brand is used to estimate children's weight. After recording anthropometric measurements for each child, the data were plotted using the IAP growth charts app (developed by MindSpace Software Technologies Private Limited, available for download at all common mobile app stores) to assess the participants' nutritional status. Section C consists of ten items for assessing the physical characteristics of a child. It consists of questions about hair, eyes, lips, gums, teeth, skin, oedema, nails, and chest. Section D has two parts to assess the associated factors for malnutrition. Part I contains a food frequency questionnaire which comprises a total of 26 items regarding dietary habits such as the habit of taking milk, curd, paneer, sprouts, butter, eggs, meat, fish, pulses, vegetables, rice, and breads consumed in the last month. Part II consists of a pre-morbid condition questionnaire which comprises a total of ten items related to family history of chronic illness, History of hospitalization of child, in the last six months, perianal itching, habit of eating chalk/mud, History of diarrhoea, cough, tobacco chewing and smoking, etc.

The tool was given to seven experts from various fields to establish the content validity. The content validity index (CVI) was calculated to be 0.84, which indicates good content validity. The inter-rater reliability for the anthropometric measurement tool and the observation of physical characteristics was found to be 0.83 and 0.9, respectively, based on Cohen's kappa test, which assessed inter-rater agreement for the same subject measured by different observers. 

Ethical clearance was obtained from the Institutional Ethics Committee with reference number: IEC/AIIMS BBSR/Nursing/2019-20/35. The objective and procedure of the study were explained to the participants in the local language. Written informed consent/assent was taken from the participants as applicable. Data were coded and analysed using IBM SPSS Statistics for Windows, Version 26 (released 2020; IBM Corp., Armonk, NY). There was no missing data. Descriptive statistics, such as frequency, percentage, mean, and standard deviation, were calculated, and inferential statistics were calculated by using the chi-square test, Fisher’s exact test, and binary logistic regression.

## Results

Out of 380 participants, 168 (44.47%) were male, and 222 (55.53%) were female. Nearly half of the children, 160 (42.10%), were between 5 and 8 years of age, while 123 (32.36%) were between 9 and 12 years of age, and 97 (25.52%) study participants were between 13 and 15 years of age. Nearly half of the participants, 185 (48.7%), and 177 (46.6%) belonged to joint and nuclear families, respectively, whereas only 18 (4.7%) belonged to extended families. One hundred and fifty (39.5%) participants had 5-7 members in their family, and only 116 (30.5%) participants had ≤4 members in their family. Two hundred and fifty-nine (68.2%) participants belong to the below poverty line (BPL) category, whereas nearly one-third, 121 (38.1%), belong to the above poverty line (APL) category (Table 1).

**Table 1:** Frequency and percentage distribution of sociodemographic variables of children (n = 380) BPL: Below poverty line, APL: Above poverty line.

Variables	Categories	Frequency n (%)
Age (years)	5–8 years	160 (42.10)
	9–12 years	123 (32.36)
	13–15 years	97 (25.52)
Gender	Male	168 (44.47)
	Female	212 (55.53)
Type of family	Nuclear	177 (46.6)
	Joint	185 (48.7)
	Extended	18 (4.7)
Total family members	≤ 4 members	116 (30.5)
	5–7 members	150 (39.5)
	≥8 members	114 (30.0)
Number of siblings	No siblings	30 (7.9)
	1–2	226 (59.5)
	3–4	99 (26.1)
	>4	25 (6.6)
Monthly family income (₹)	<5000	43 (11.3)
	5000–10000	209 (55.0)
	10000–20000	119 (31.3)
	>20000	9 (2.4)
Standard of living	BPL	259 (68.2)
	APL	121 (31.8)
Father’s education	No formal education	91 (23.9)
	Up to primary	110 (28.9)
	Secondary education	144 (37.9)
	Graduation & above	35 (8.7)
Mother’s education	No formal education	89 (23.4)
	Up to primary	119 (31.3)
	Secondary education	146 (38.4)
	Graduation & above	26 (6.8)

The mean weight of the participants was 30±12.336 kg, ranging from 9.70 to 72 kg, whereas the mean height of the participants was 133.19±16.64 cm, which ranged from 97 to 167.5 cm. The mean BMI of the participants was 16.26 ± 3.84 kg/m^2^, with a range of 12.75-27.58 kg/m² (Table 2).

**Table 2:** Mean, standard deviation and range of anthropometry measurements of study subjects (n=380)

Variables	Mean ± SD	Range (Min–Max)
Weight (kg)	30.00 ± 12.33	9.70 – 72.00
Height (cm)	133.19 ± 16.64	97.00 – 167.50
BMI (kg/m²)	16.27 ± 3.84	12.75 – 27.58

As shown in Table 3, 222 (58.7%) of the study participants had normal nutritional status, whereas 158 (41.3%) participants were malnourished. Out of which 54 (14.2%) were underweight, 49 (12.8%) were overweight, and only 20(5.2%) were obese. Moreover, 10 (2.6%) were stunted, 16 (4.2%) were underweight and stunted, and 9 (2.3%) were overweight and stunted.

**Table 3:** Distribution of nutritional status among study participants (n = 380)

Nutritional Status Category	Frequency (n)	Percentage (%)
Normal	222	58.7
Malnourished (Total)	158	41.3
Underweight	54	14.2
Overweight	49	12.8
Obese	20	5.2
Stunted	10	2.6
Underweight and Stunted	16	4.2
Overweight and Stunted	9	2.3

Age-wise analysis of malnutrition, shown in Table 4, indicates that all categories of malnutrition were the highest among 5 to 8-year-old participants. In the age group 5-8 years, 32 (20.25%) were underweight, 5 (3.16%) were stunted, 19 (12.02%) were overweight, 9 (5.69%) were obese, 4 (1.26%) were overweight and stunting, and 6 (3.79% ) were underweight and stunting.

**Table 4:** Age-wise categories of malnutrition as per the IAP growth charts app (n=158) IAP growth charts app (Developed by MindSpace Software Technologies Private Limited, available for download at all common mobile app stores).

Age group (years)	Underweight n (%)	Stunting n (%)	Overweight n (%)	Obese n (%)	Overweight & Stunting n (%)	Underweight & Stunting n (%)	Total n (%)
5–8	32 (20.25)	5 (3.16)	19 (12.02)	9 (5.69)	4 (2.53)	6 (3.79)	75 (47.46)
9–12	15 (9.49)	4 (2.53)	18 (11.39)	9 (5.69)	2 (1.26)	4 (2.53)	52 (32.92)
13–15	7 (4.43)	1 (0.63)	12 (7.59)	2 (1.26)	3 (1.89)	6 (3.79)	31 (19.62)
Total	54 (34.17)	10 (6.34)	49 (31.01)	20 (12.65)	9 (5.69)	16 (10.14)	158 (100.0)

To identify the factor associated, a Chi-square test was performed by considering two categories, i.e., malnourished (158) and non-malnourished (222). The factors associated with malnutrition includes: type of family (χ2 (2) = 8.396, p=0.015), number of family member (χ2 (2)= 9.170, p=0.010), income of the family (χ2 (3)= 24.82, p=0.001), usage of closed latrine (χ2 (1) =11.607, p=0.006), consumption of filtered water (χ2 (1) = 7.933 p=0.005), Mother’s education (χ2(3)= 12.882, p=0.012), Health check-ups available in school (χ2 (1)= 7.523 p=0.006). Furthermore, there is no significant association between malnutrition and the number of siblings, source of drinking water, father’s occupation, mother’s occupation, gender, or age

As shown in Table 5, to identify the factors contributing to malnutrition, binary logistic regression was performed. Significant contributing factors included: large family size (>4 members): OR=2.019, mother’s education (No):OR = 2.045, duration of the disease (>3 months):OR = 2.454, lack of filtered water (yes):(OR = 1.883), consumption of pulses (No):OR = 2.048, consumption of meat (No):OR = 2.372, father's education (OR = 2.302) and family history of disease (Yes): OR = 1.934, availability of health checkup in school (No):OR = 1.776, availability of closed latrine (No):OR = 2.082

**Table 5:** Binary logistic regression results for contributing factors of malnutrition (n = 380) * P-value < 0.05 is significant; CI: confidence interval.

S. No.	Variable	Category (Reference)	OR	95% CI	χ²	p-value
1	Family size	>4 members (<4)	2.019	1.269–3.212	8.943	0.003*
2	Availability of water filtration	No (Yes)	1.883	1.209–2.933	7.933	0.005*
3	Father’s educational status	No formal education (Formal)	2.302	1.265–3.307	8.69	0.003*
4	Mother’s educational status	No formal education (Formal)	2.045	1.426–3.717	8.69	0.003*
5	Family history of disease	Yes (No)	1.934	1.082–3.457	5.062	0.024*
6	Duration of illness	>3 months (< 3months)	2.454	1.469–4.099	12.16	0.0004*
7	Availability of closed latrine	No (Yes)	2.082	1.362–3.184	11.61	0.0006*
8	Health checkups in school	No (Yes)	1.776	1.176–2.082	7.523	0.003*
9	Consumption of pulses	No (Yes)	2.548	1.360–4.775	8.941	0.003*
10	Consumption of meat	No (Yes)	2.372	1.335–4.215	9.014	0.03*

## Discussion

Nutritional assessment plays a vital role in early identification of malnutrition and its contributing factors among school-going children and adolescents. The present study revealed that the prevalence of malnutrition among children aged 5-15 years in selected villages of Khurda district of Odisha was 41.3%, with 13.9% under weight children. These findings are consistent with several national and global reports indicating that undernutrition among school-age children remains a significant public health concern.

Globally, the United Nations Children's Fund (UNICEF) estimates that the prevalence of underweight among children aged 5-19 years is approximately 9.2%, although South Asia continues to bear a disproportionately higher burden compared with other regions [11]. The Comprehensive National Nutrition Survey reported that about 35% of Indian children aged 5-9 years were underweight, indicating a substantial burden of undernutrition among school-age children [12]. These figures support the relatively high prevalence observed in the present study. Evidence from Bangladesh and Nepal continues to report notable levels of undernutrition, with about 21% of children under five classified as underweight, indicating continuing nutritional vulnerability in the region [13]. A cross-sectional study conducted among school children in Rajasthan reported an overall malnutrition prevalence of 24.17%, with 19.72% of the children being underweight, highlighting the persistent burden of undernutrition among school-age children in India [14]. Similarly, another study among school-going children reported a high prevalence of malnutrition (around 51%) in children aged 6-16 years, suggesting that nutritional deficiencies remain widespread even among school-age populations [15]. 

Odisha continues to face significant nutritional challenges, with around 29% of children under five reported as underweight in the state, reflecting persistent regional disparities in nutritional outcomes [6]. A community-based study in urban slum children in Odisha reported high levels of undernutrition with considerable proportions of underweight (55.3%), wasting (75%) and stunting (42%), reflecting the continued vulnerability of children in socioeconomically disadvantaged settings [16]. Variations in prevalence may be attributed to differences in age groups studied, socioeconomic conditions, dietary practices, environmental sanitation, and accessibility of nutrition programmes.

In the present study, the prevalence of stunting among children aged 5-15 years was 2.6%, which is considerably lower compared to several other studies. National Family Health Survey-5 reports approximately 35.5% of children under five years of age in India are stunted, indicating that, despite improvements over recent decades, undernutrition continues to pose a major challenge. State-level data show considerable variation, with Odisha reporting about 31% stunting among children under five years, highlighting persistent regional disparities influenced by poverty, dietary patterns, sanitation, maternal education, and access to healthcare services [12].

The Comprehensive National Nutrition Survey further demonstrates that nutritional deficits extend beyond early childhood. The survey reported measurable levels of stunting among school-age children (5-9 years) and adolescents (10-19 years), suggesting that growth faltering often persists into later childhood and adolescence when nutritional deficiencies remain unaddressed [6,17]. These findings emphasise the importance of sustained nutritional interventions beyond the first five years of life.

Several regional studies across India have reported varying prevalence of stunting among school-age children and adolescents, often influenced by socio-economic status, dietary intake, and environmental conditions [14,15]. Higher prevalence has frequently been observed among children residing in rural areas, urban slums, and socio-economically disadvantaged populations [18-20]. Differences in age groups studied, assessment methods, and geographic contexts may partly explain variability in reported prevalence rates.

Compared with these national and regional findings, the prevalence of stunting observed in the present study appears relatively lower. This discrepancy may be attributed to differences in study population characteristics, age range (5-15 years), the study being conducted during the COVID-19 period, and potential benefits from ongoing public health nutrition programs, such as the Integrated Child Development Services (ICDS), the Mid-Day Meal Scheme, and the Poshan Abhiyan. These programmes may contribute to improved nutritional awareness, food security, and growth outcomes among school-age children. Nevertheless, as stunting reflects long-term nutritional deprivation, continued multi-sectoral interventions addressing diet, sanitation, education, and socio-economic determinants remain essential to reduce its burden.

Parental education emerged as a strong determinant of child nutritional status. Children of non-formally educated mothers and fathers were 2.04 times and 2.302 times more likely, respectively, to be malnourished compared with those of educated parents. A study conducted in West Bengal similarly reported that educated mothers were 1.4 times less likely to have underweight children [10]. Limited awareness of nutritional requirements among less educated parents may contribute to inappropriate feeding practices and poor child health outcomes. Family environment plays a critical role in child growth and nutritional outcomes. Supporting evidence from Uttar Pradesh indicated that mothers with less than six years of schooling were 3.81 times more likely to have malnourished children [21].

Family size emerged as a strong predictor of malnutrition. Children from families with more than four members were 2.09 times more likely to be malnourished compared to those from smaller families. Similar findings were reported in another study in West Bengal, which suggested that larger family size may strain household resources, thereby affecting children’s nutritional intake [20]. Sanitation practices were significantly associated with nutritional status. Availability of closed latrines reduced the risk of malnutrition by 2.08 times. These findings are consistent with previous research indicating that open defecation increases the likelihood of undernutrition by approximately 2.2 times [22], emphasizing the importance of improved sanitation in preventing childhood malnutrition.

The availability of regular health checkups in schools was a key determinant of the lower prevalence of malnutrition in this study, with children receiving routine health assessments being 1.7 times less likely to be malnourished. However, another study reported contrasting findings; mere proximity to health facilities was not significantly associated with stunting, underweight, or thinness, suggesting that utilization and quality of healthcare services may be more important than accessibility alone [23]. Regular screening can facilitate early identification of nutritional deficiencies, allowing timely intervention, parental counseling, and appropriate referral. This preventive approach may help reduce the progression of nutritional deficits into overt malnutrition. Family health conditions also influenced child nutritional status, with a family history of disease increasing the risk of malnutrition by 1.9 times, possibly because illness within the family may reduce caregiving capacity or financial resources available for child nutrition [24]. Similarly, a history of chronic disease in the family increased malnutrition risk by 2.4 times, which may reflect long-term socioeconomic and caregiving challenges associated with chronic illness.

Dietary intake of specific foods such as milk, pulses, green leafy vegetables, fish, meat, fruits, and curd further influenced nutritional outcomes. Children who did not consume butter, paneer, and meat were 1.54, 1.73, and 2.37 times more likely to develop malnutrition, respectively. Conversely, children who consumed pulses, vegetables, fruits, sprouts, and bread had significantly lower risks of malnutrition. Lack of consumption of sprouts and bread was more likely to increase the risk by 2.08 and 2.31 times, respectively. These findings are supported by a study conducted in Nepal, which showed that vegetable consumption was associated with lower underweight prevalence (OR = 0.49). Similarly, another study reported that consumption of milk and milk products reduced the risk of stunting (OR = 0.616) and underweight (OR = 0.72), whereas meat consumption was associated with a reduced likelihood of underweight [21]. Adequate dietary diversity is essential for maintaining proper growth and preventing nutritional deficiencies in school-aged children. 

Strengths of the study include (a) Community-based design: enhances generalizability to the rural population, (b) Simple random sampling: minimizes selection bias, (c) Use of a standardized tool (IAP growth charts): ensures objective and replicable classification of nutritional status, and (d) Comprehensive analysis: the use of both bivariate (Chi-square) and multivariate (logistic regression) analysis allow for the identification of independent association. 

The following were the limitations of the study: (a) Due to the COVID-19 pandemic, there was a sudden change in the life styles of children; socioeconomic status of the family could be the reason for the high prevalence of malnutrition, (b) The study was limited only to two villages of Khordha district, which could be less for generalizing the study findings, (c) This is a cross-sectional study that can only identify associations, not causation, (d) The study used a food frequency questionnaire and asked about past illnesses (last six months), both of which are susceptible to recall bias, especially in children, (e) Responses about hygiene practices (latrine use, water filtration) may be influenced by what participants think the researcher wants to hear, (f) Exclusion of children with chronic illness may underestimate malnutrition prevalence.

## Conclusions

The overall prevalence of malnutrition was 41.3 %, and all categories of malnutrition were quite high between five and eight years of age. The prevalence of malnutrition was lower in older age groups. Further, the study found that larger family size, lack of parental education, poor hygiene (lack of closed latrine/filtered water), lack of access to filtered water, and inadequate dietary intake were key predictors and significant risk factors for malnutrition. The recommendation could be tailored to these findings.
